# Direct Facilitatory Role of Paragigantocellularis Neurons in Opiate Withdrawal-Induced Hyperactivity of Rat Locus Coeruleus Neurons: An In Vitro Study

**DOI:** 10.1371/journal.pone.0134873

**Published:** 2015-07-31

**Authors:** Ayat Kaeidi, Hossein Azizi, Mohammad Javan, S. Mohammad Ahmadi Soleimani, Yaghoub Fathollahi, Saeed Semnanian

**Affiliations:** Department of Physiology, Faculty of Medical Sciences, Tarbiat Modares University, PO Box 14115–331, Tehran, Iran; University of Leicester, UNITED KINGDOM

## Abstract

Studies have shown that following opiate withdrawal, the spontaneous discharge rate of locus coeruleus (LC) neurons remarkably increases. Combination of intrinsic mechanisms with extrinsic excitatory modulations mediates the withdrawal-induced hyperactivity of LC neurons. The nucleus paragigantocellularis (PGi) provides the main excitatory inputs to LC and plays a pivotal role in opiate withdrawal. In the present study the direct facilitatory role of PGi on opiate withdrawal-induced hyperactivity of LC neurons was investigated using a newly developed brain slice, containing both LC and PGi. HRP retrograde neuronal tracing was used to verify the existence of both LC and PGi neurons in the developed slice. The spontaneous discharge rate (SDR), resting membrane potential (RMP) and spontaneous excitatory post-synaptic currents (sEPSCs) were recorded in LC neurons using whole cell patch clamp recording. Results showed that the net SDR and the net RMP of LC neurons in slices containing both LC and PGi neurons are significantly higher than slices lacking intact (uncut) PGi inputs. Also, the frequency of sEPSCs in those LC neurons receiving PGi inputs significantly increased compared to the slices containing no intact PGi inputs. Altogether, our results propose that increase in PGi-mediated excitatory transmission might facilitate the opiate withdrawal-induced hyperactivity of LC neurons.

## Introduction

Locus coeruleus (LC) nucleus, which is bilaterally located on the floor of the fourth ventricle, is the largest cluster of noradrenergic neurons in brain stem [1,2]. This region expresses a high density of opioid receptors and because of its relatively homogenous structure, serves as a good model for studying opiate actions [3]. Different in vivo and in vitro investigations have shown that acute morphine administration decreases LC neuronal activity, whereas these neurons undergo significant tolerance to opiate effects during chronic opiate exposure [4]. This finding has been previously confirmed by the return of LC neuronal firing rate toward the pretreatment levels [5]. In addition, following opiate withdrawal, the spontaneous firing rate of LC neurons increases dramatically above the normal level [5,6].

Furthermore, different studies have shown that increment in LC neuronal activity following opioid withdrawal might be mediated (to some extent) through intrinsic mechanisms within the LC neurons. Different electrophysiological studies have reported the hyperactivity of LC neurons following naloxone-induced withdrawal in brain slices taken from chronically morphine treated rats [7,8]. In addition, chronic exposure to opiate drugs resulted in diversified neurochemical changes in LC neurons, which were manifested by enhancement of G-protein transduction systems [9], adenylyl cyclase activity [10], cAMP-dependent protein kinase activity [11] and induction of phosphorylated proteins [12–14] such as, cAMP response element-binding protein (CREB) and tyrosine hydroxylase (TH) [15].

However, aside from the intrinsic properties of LC neurons, other mechanisms responsible for the increased LC neuronal activity following opiate withdrawal have been proposed to be mediated through extrinsic signaling pathways. Several in vitro studies have not reported any increment in spontaneous neuronal activity of LC neurons in brain slices of chronically morphine-treated rats [16–18]. In addition, Akaoka and Aston-Jones (1991) have previously shown that intra-LC microinjection of naloxone does not activate LC neurons in morphine dependent rats, whereas systemic administration of naloxone strongly increases LC neuronal activity. They have proposed the increased excitatory amino acid neurotransmission within LC region as the responsible factor for this naloxone-induced hyperactivity of LC neurons [12].

Moreover, several investigations have indicated that the excitatory afferents from other brain regions such as medulla may play a critical role in withdrawal-induced hyperactivity of LC neurons [4,19]. Different anatomical and physiological investigations of afferents to LC have revealed that this nucleus receives afferents from only a restricted number of brain loci. Of them the nucleus paragigantocellularis (PGi) (located in the rostroventrolateral medulla, RVLM) has been suggested as one of the major brain nuclei sending excitatory (glutamatergic) afferents to LC region [20]. Previous studies have shown that approximately 73% of LC neurons could be synaptically activated by applying low intensity single-pulse electrical stimulations to PGi neurons [21], whereas, opiate withdrawal-induced hyperactivity of LC neurons remarkably decreases following lesions of PGi nucleus [22].

On the basis of the above mentioned background, the present study has been designed to postulate the direct facilitatory role of PGi in opiate withdrawal-induced hyperactivity of LC neurons using a newly developed slice preparation containing both LC and PGi neurons.

## Materials and Methods

### Ethics statement

Attention was paid to minimize animal suffering during the entire experimental period. All procedures were performed according to the ethical guidelines of Faculty of Medical Sciences, Tarbiat Modares University based on the United States NIH Guide for the Care and Use of Laboratory Animals (publication no. 85–23). All performed experimental protocols in this study were approved by the Ethical Committee of Faculty of Medical Sciences, Tarbiat Modares University.

### Animals

Male Wistar rats (2–3 weeks old for electrophysiological and 10–12 weeks for neuronal tracing experiments) were used in this study. Rats were housed in Plexiglas breeding cages with woodchip bedding and free access to food and water. Animals were kept in a colony room with constant temperature and on 12 h light/dark cycles (the light period started at 7 a.m.).

### Drugs

The main chemical substances used in this study were as follows: morphine sulphate (Temad Co., Iran), naloxone hydrochloride (Sigma Aldrich, USA), horseradish peroxidase (HRP) type VI (Sigma Aldrich, USA), bicuculline (Sigma Aldrich, USA), chloral hydrate (Merck, Germany), diethyl ether (Merck, Germany) and tetramethylbenzidine (TMB) (Sigma Aldrich, USA).

### Retrograde HRP neuronal tracing and histochemistry

Horseradish peroxidase (HRP) neuronal tract-tracing is a method of intra-axonal transport of HRP, where its cellular uptake occurs in somata, dendrites, as well as axon terminals through endocytosis [23] and it can be transported in both anterograde and retrograde directions [24, 25]. This technique is methodologically easy, can be rapidly performed, reaction products can be visualized by simple histochemical processes and the results may be visible under the light microscope [23].

In our study, in order to perform retrograde HRP neuronal tracing, initially rats were deeply anesthetized by chloral hydrate (400 mg/kg, i.p.). Thereafter, 0.1 μl of 10% HRP (dissolved in sterile saline) was injected slowly into the LC via a long-shank glass micropipette (connected to a 1-μl Hamilton syringe), which was left in the site for 5 min to prevent any backflow of the injected HRP. After that, the pipette was smoothly removed and the incision site was carefully sutured with all aseptic precautions.

After 24–36 h, rats were deeply anesthetized again with aforementioned maneuver and transcardially perfused with 250 ml PBS (37°C) followed by 50–100 ml of fixative solution (1% paraformaldehyde and 1.25% glutaraldehyde in 0.1 M phosphate buffer at pH 7.4, 4°C). Brains were carefully removed from the skull and cryoprotected by overnight soaking in PBS (0.1 M) containing sucrose (30%). The tissue blocks were serially sectioned into 40 μm-thick slices using a freezing microtome (Histo-Line Laboratories, Italy). Finally, the prepared sections were processed for HRP reaction by TMB method [26] and counterstained with 0.1% neutral red.

### Slice preparation

**Fig 1:**
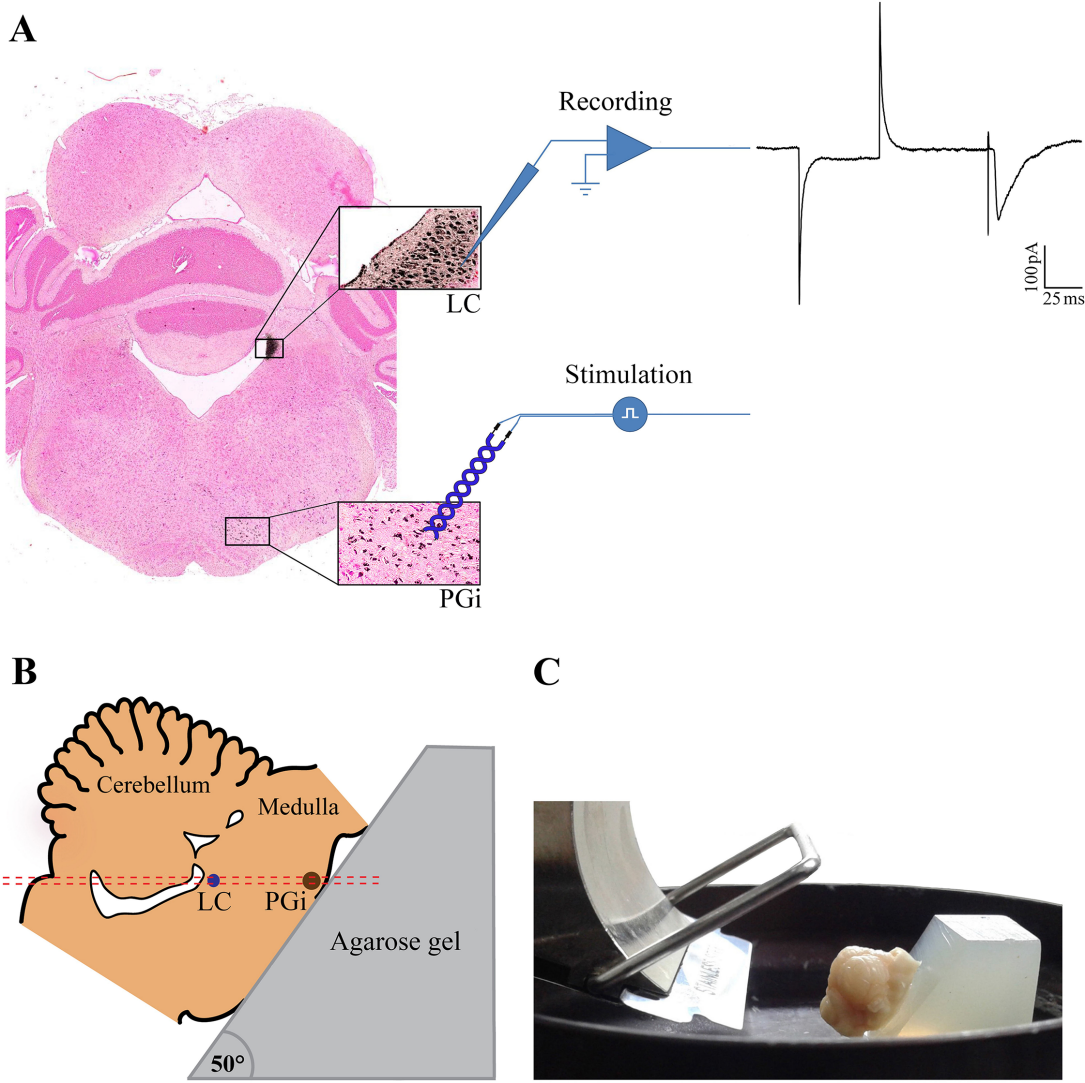
Photomicrograph of a typical OTH brain section. Photomicrograph shows retrogradely labeled PGi neurons in the rostra1 ventral medulla of a rat which received intra-LC microinjection of HRP (A, left side). Insets illustrate high magnification of LC and PGi regions. Following a single pulse electrical stimulation of PGi, an evoked EPSC was recorded from LC neuron (A, right side). The schematic representation of cutting stage orientation for preparation of OTH brain slices. The tissue block containing LC and PGi were mounted on the wedge-shaped agarose gel with an angle of 50°. The red dashed line shows the cutting direction (B). Picture shows the blade direction and the way through which tissue block was mounted on cutting stage (C). OTH: oblique to horizontal; LC: locus coeruleus; PGi: paragigantocellularis; HRP: horseradish peroxidase; EPSC: excitatory post synaptic current.

In this study, three forms of brain slices were prepared: horizontal (HZ) slices (containing LC but not PGi neurons), oblique to horizontal (OTH) slices (containing both LC as well as PGi neurons, parallel to the LC-PGi connecting bundles) and oblique to horizontal-cut (OTH-cut) slices (similar to OTH slices containing both LC and PGi neurons, but connecting area between LC and PGi was linearly cut). For these, rats were deeply anaesthetized by diethyl ether and decapitated. Brains were quickly removed from the skull and trimmed in ice-cold (1–4°C), low-calcium sucrose-based artificial cerebrospinal fluid (sucrose-aCSF) containing (in mM) sucrose 213, KCl 2.6, CaCl_2_ 0.1, MgCl_2_ 2, NaHCO_3_ 26, NaH_2_PO_4_ 1.23, L-ascorbic acid 0.4, D-glucose 2 (290–310 mOsmol/L, pH 7.3–7.4 when bubbled with 95% O_2_, 5% CO_2_). For preparing HZ slices, a block of tissue containing LC was glued to the cutting stage of a vibratome (1000 Plus Sectioning System, Vibratome, USA) with the dorsal side up. For preparing OTH slices, the ventral side of a tissue block containing both LC and PGi nuclei were mounted on the steep surface of a wedge-shaped agarose gel piece which was glued to the cutting stage of the vibratome. The slope of this agarose wedge was 50° in a way that LC, PGi and the vibratome blade were aligned in the same orientation (Fig 1B and 1C). Finally, for all forms of slices (HZ, OTH and OTH-cut), 2–3 brain sections of 400 μm thicknesses were provided at 1–4°C. The procedure of preparing OTH slices was the same for both electrophysiological study and HRP neuronal tracing.

The slices were first incubated in a holding chamber with standard aCSF containing (in mM) NaCl 125, KCl 3, NaH_2_PO_4_ 1.25, NaHCO_3_ 25, CaCl_2_ 2, MgCl_2_ 1.3, L-ascorbic acid 0.4 and D-glucose 10, saturated with 95% O_2_ and 5% CO_2_ for 30 to 40 min at 35°C. Osmolarity was maintained between 290 and 310 mOsmol/L and pH between 7.3 and 7.4. Slices were then kept at room temperature (~25°C) in the same chamber until the recording time. Then all the slices were transferred to the recording chamber mounted on a fixed-stage upright microscope (Axioskop 2 FS, Carl Zeiss, Germany) and were made fixed there under nylon strings attached to a U-shaped platinum frame. All brain slices were continuously perfused with the standard aCSF at a flow rate of 1 to 2 ml/min. LC neurons were visualized using an IR-CCD camera (IR-1000, USA) with 10X and 40X water immersion objective lenses.

### Whole-cell patch-clamp recording

The patch electrodes were made from borosilicate glass pipettes (1.5 mm outer diameter, GC150-11; Harvard Apparatus, UK) with a programmable puller (P-97, Sutter Instrument, USA). The tip resistance of the electrode was 3 to 7 MΩ when filled with intracellular solution containing (in mM) potassium gluconate 120, NaCl 6, CaCl_2_ 1, MgCl_2_ 2, MgATP 2, NaGTP 0.5, phosphocreatine Na_2_ 12, EGTA 5 and HEPES hemisodium 10 (pH 7.3 as adjusted with KOH; osmolarity 285–290 mOsmol/L). The LC neurons were preselected according to the IR-DIC image. After establishment of the cell attached configuration (3–8 GΩ seal resistance), the whole cell mode was established with a brief negative pressure pulse. Recording was started at least 5 min after the rupture of the patch membrane to stabilize the intracellular milieu. Access resistance of < 20 MΩ was considered acceptable and was monitored periodically throughout the experiment. The experiment was terminated if the access resistance changed more than 15%. Whole-cell current clamp recordings were acquired with a MultiClamp 700B amplifier and pClamp 10 software (Molecular Devices, USA), with a 3 kHz low-pass Bessel filter, and digitized at 10 kHz using a Digidata 1440A data acquisition system (Molecular Devices, USA).

Spontaneous EPSCs (sEPSCs) were recorded in voltage clamp mode at a holding potential of -70 mV and isolated using GABA_A_ receptor antagonist (bicuculline, 20 μM). The amplitude and the frequency of each sEPSC were measured. Currents with peak amplitude smaller than 8 pA (depending on the basal noise level of the recording) were excluded from analysis. All the sEPSCs were recorded 5 min before and after naloxone application (1 μM, 5 min).

### Experimental groups

All the rats were grouped as, rats without any treatment (naïve, untreated, non-dependent, control) and rats chronically treated with morphine (morphine-dependent, 20 mg/kg, daily, i.p. for consecutive 7 days).

Resting membrane potential (RMP), frequency of spontaneous discharge rates (SDR) and the amplitude as well as frequency of sEPSCs, in LC neurons, were evaluated in horizontal (HZ), oblique to horizontal (OTH) and oblique to horizontal-cut (OTH-cut) brain slices prepared from the rats of two main above mentioned groups.

In our study, it is mentionable that, in another experimental group, the acute effect of morphine on RMP and SDR was measured in brain slices taken from non-dependent rats (acute morphine group). For studying the acute effect of morphine, all slices taken from non-dependent rats were incubated and perfused with morphine (5 μM) during 60 to 90 min of the experiment. The slices taken from morphine-dependent rats were continuously bathed in morphine (5 μM) during the whole experimental period.

In addition, for all OTH brain slices, only LC neurons having monosynaptic connection with PGi afferents (as evidenced by recording the evoked EPSC from LC neurons in response to single pulse electrical stimulation of PGi region) were included in the experiments (Fig 1A).

Moreover, in order to induce withdrawal in LC neurons of brain slices taken from chronically morphine treated rats, naloxone (1 μM) was superfused after 5 min baseline recording.

Furthermore, in order to investigate the effect of PGi excitatory afferents on naloxone-induced hyperactivity of LC neurons in chronically morphine treated rats, kynurenic acid (Kyn, 500 μM, an excitatory amino acid antagonist) was used.

### Data analysis

All values are expressed as mean ± SEM. Electrophysiological data were analyzed off-line using Clampfit software (pClamp 10; Molecular Devices, USA) and statistical analysis was performed using GraphPad Prism version 6.01 for Windows (GraphPad Software, USA). Results were compared before and after naloxone administration using paired Student's t-test or a one-way ANOVA for multiple group comparisons. Tukey’s post hoc test was used following ANOVA to test significance among different groups.

The net increment in electrophysiological indices was calculated by subtracting the values of pre and post naloxone administration. P < 0.05 was considered statistically significant.

## Results

### Slice preparation and histology

As shown in Fig 1A, intra-LC microinjections of HRP retrogradely labeled the ipsilateral PGi neurons in the rostra1 ventral medulla. This figure indicates that both LC and PGi neurons were present in the newly developed OTH brain slices. Furthermore, the cutting method is shown in Fig 1B and 1C.

### Spontaneous discharge rate and resting membrane potential in naïve group

**Fig 2:**
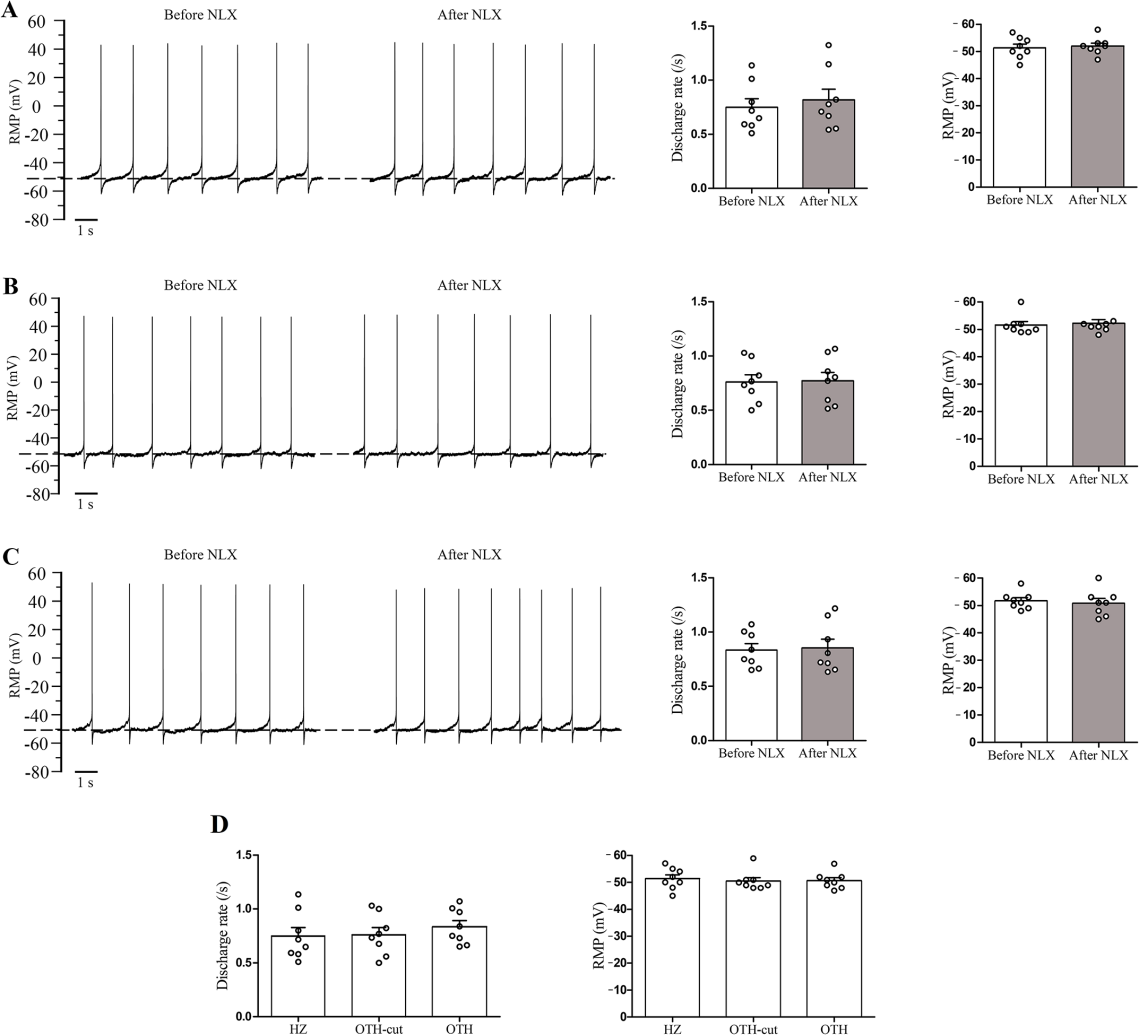
SDR and RMP of LC neurons in naïve (non-dependent) rats. Representative traces show the spontaneous discharge activity of three LC neurons before and 5 min after naloxone superfusion (1 μM) in HZ (A), OTH-cut (B) and OTH (C) brain slices. Histograms (A–C) indicate the mean RMP and frequency of SDR recorded before and after naloxone application. Note that naloxone did not alter the SDR and RMP of LC neurons in naïve rats. The dashed line represents the RMP. Summary data showing the mean SDR and RMP in LC neurons of all three slice forms in naïve rats, which were not significantly different among themselves (D). Data are expressed as mean ± SEM, n = 8 in each type of brain slice. Data were analyzed using paired Student’s t-test (A–C) and one-way ANOVA followed by Tukey’s post hoc test (D). SDR: spontaneous discharge rates; RMP: resting membrane potential; NLX: naloxone; LC: locus coeruleus; HZ: horizontal; OTH-cut: oblique to horizontal-cut; OTH: oblique to horizontal.

In naïve rats, for each form of slice preparation, 8 neurons were investigated from separate slices. Only neurons with stable spontaneous discharge frequency and membrane potential were included in data analysis. In all forms of slices, there were no significant differences among the SDR of LC neurons (the mean SDR were 0.74 ± 0.1, 0.76 ± 0.06 and 0.83 ± 0.05/s for HZ, OTH-cut and OTH brain slices, respectively; Fig 2D, left side). Similarly, RMPs were also close together for all brain slice forms (-53.38 ± 1.4, -51.63 ± 1.3 and -51.8 ± 1 mV for HZ, OTH-cut and OTH brain slices, respectively; Fig 2D, right side). In addition, the effect of naloxone was tested in all cells of this group, where no significant change was observed in RMP as well as in SDR (Fig 2A–2C).

### Spontaneous discharge rate and resting membrane potential in acute morphine group

**Fig 3:**
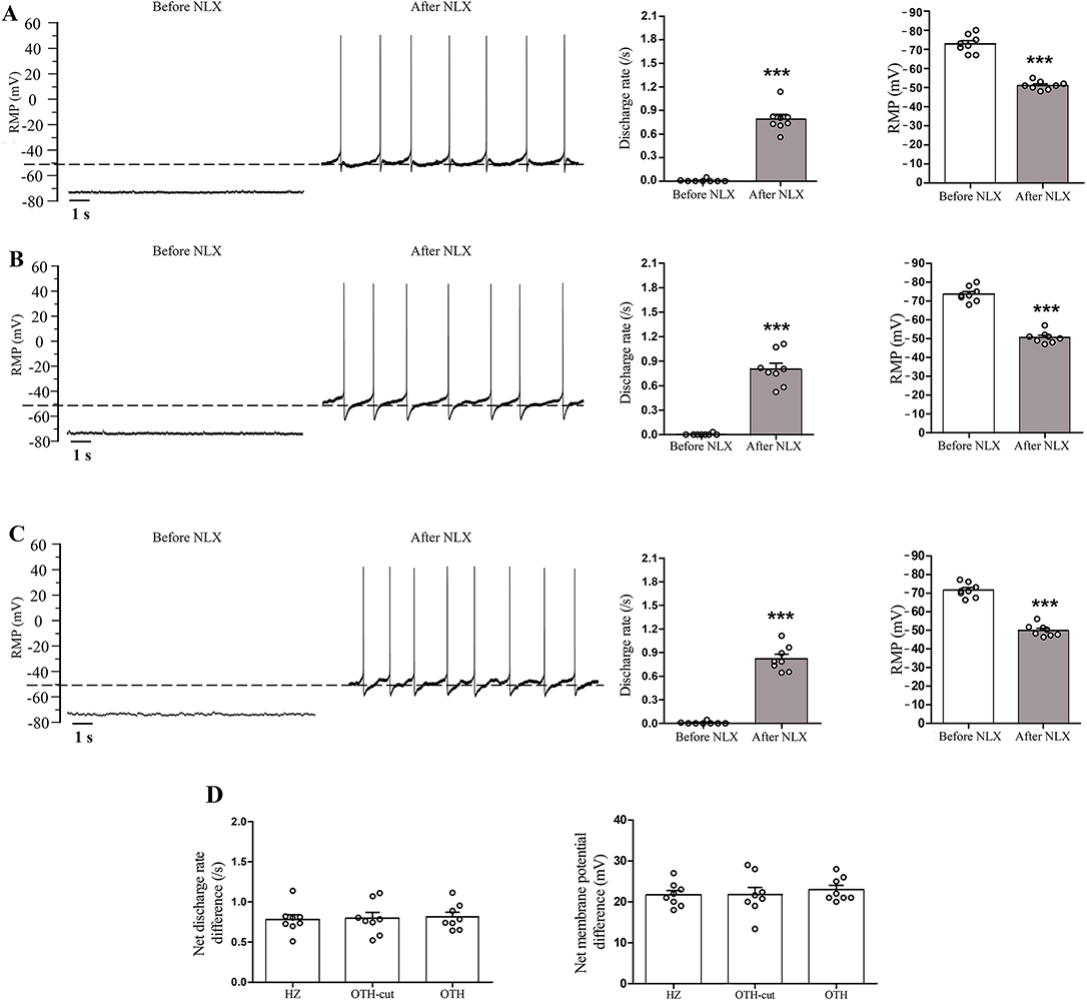
Effect of acute morphine application on SDR and RMP of LC neurons in naïve rats. Representative traces show the RMP and SDR of three LC neurons before and 5 min after naloxone superfusion (1 μM) in HZ (A), OTH-cut (B) and OTH (C) brain slices (slices taken from naïve animals were incubated with 5 μM morphine for 60 to 90 min before naloxone application). Histograms (A–C) indicate the mean RMP and frequency of SDR recorded before and after naloxone application. Acute incubation of brain slices with 5 μM morphine led to significant decrement in RMP and suppression of the SDR of LC neurons in non-dependent animals. Note that naloxone returned SDR and RMP values to the baseline levels in naïve animals. It should be noted that the net effect of naloxone on RMP and SDR was similar among the three forms of brain slices (D). The dashed line represents the RMP after naloxone superfusion. Data are expressed as mean ± SEM, n = 8 in each type of brain slice, *** P < 0.001, compared to before naloxone application. Data were analyzed using paired Student’s t-test (A–C) and one-way ANOVA followed by Tukey’s post hoc test (D). RMP: resting membrane potential; SDR: spontaneous discharge rates; NLX: naloxone; LC: locus coeruleus; HZ: horizontal; OTH-cut: oblique to horizontal-cut; OTH: oblique to horizontal.

In the present study, acute administration of 5 μM morphine for 60 to 90 min led to significant decrement in RMP of LC neurons in non-dependent animals (-73 ± 4.7, -74 ± 3.9 and -72 ± 4.1 mV for HZ, OTH-cut and OTH brain slices, respectively; Fig 3A–3C). Furthermore, SDR was suppressed in LC neurons of all slices in the presence of morphine (Fig 3A–3C). On the other hand, when naloxone (1 μM) was applied following acute morphine administration, SDR as well as RMP were returned to the baseline levels in the non-dependent animals (0.78 ± 0.16, 0.82 ± 0.18 and 0.8 ± 0.21/s; -51 ± 2.2, -50.6 ± 3.1 and -50.7 ± 4 mV for HZ, OTH-cut and OTH brain slices, respectively; Fig 3A–3C). These findings suggest that RMP and SDR of LC neurons did not change by naloxone administration after 60 to 90 min morphine superfusion compared to the baseline in all forms of brain slices taken from naïve animals. It should be noted that, no significant differences were observed in the net effect of naloxone on RMP and SDR in three forms of brain slices (Fig 3D).

### Spontaneous discharge rate and resting membrane potential in morphine-dependent group

**Fig 4:**
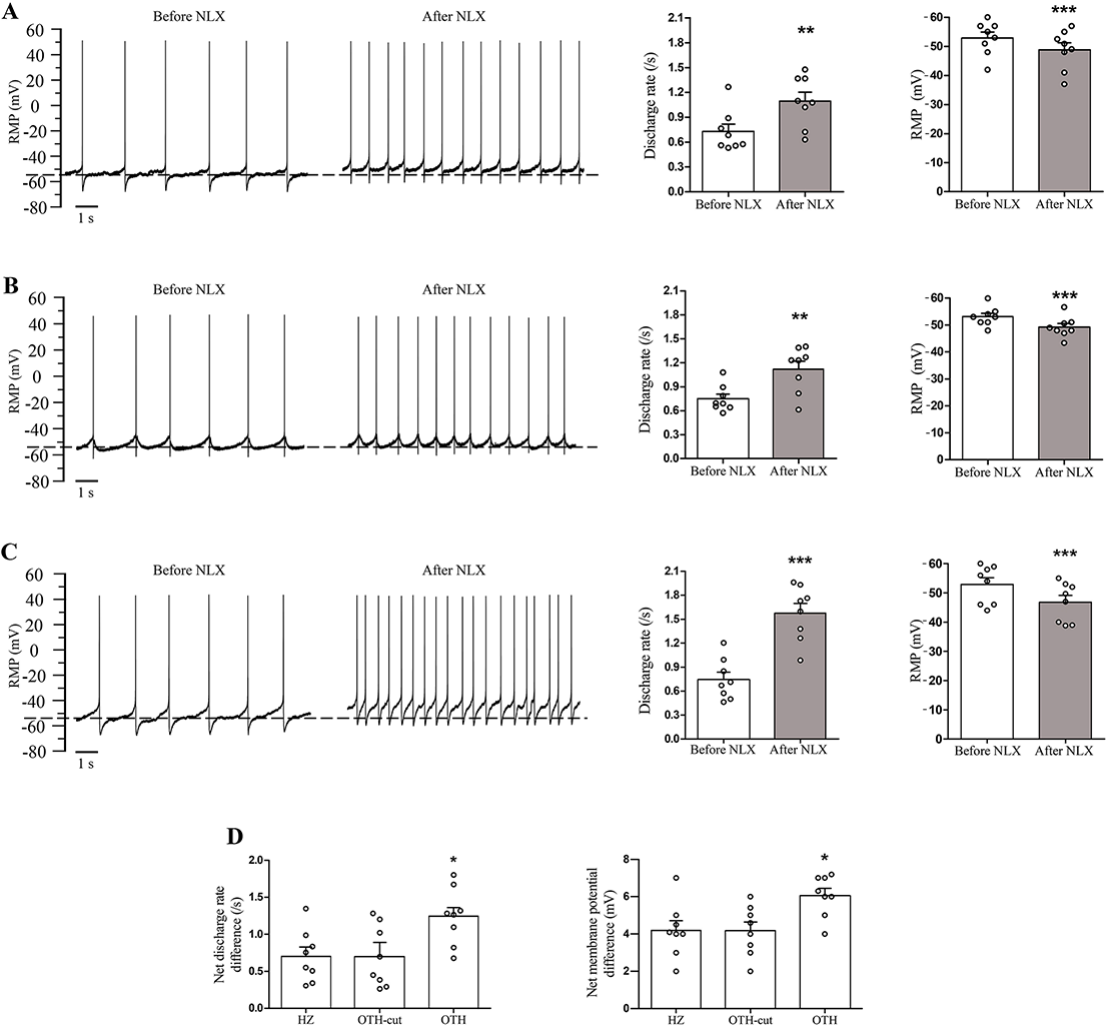
Effect of naloxone on SDR and RMP of LC neurons in morphine-dependent rats. Representative traces show the spontaneous discharge activity and the RMP of three LC neurons before and 5 min after naloxone superfusion (1 μM) in HZ (A), OTH-cut (B) and OTH (C) brain slices. The slices were bathed in 5 μM morphine. Histograms indicate the mean RMP and frequency of SDR recorded before and after naloxone application. Naloxone significantly increased the SDR and RMP of LC neurons in all form of brain slices. It should be noted that the net (not the mere) effect of naloxone on RMP and SDR was significantly higher in OTH brain slices than those of HZ and OTH-cut preparations taken from morphine dependent rats (D). Data are expressed as mean ± SEM, n = 8 in each type of brain slice, * P < 0.05, ** P < 0.01 and *** P < 0.001 compare to HZ and OTH-cut. Data were analyzed using paired Student’s t-test (A–C) and one-way ANOVA followed by Tukey’s post hoc test (D). RMP: resting membrane potential; SDR: spontaneous discharge rates; NLX: naloxone; LC: locus coeruleus; HZ: horizontal; OTH-cut: oblique to horizontal-cut; OTH: oblique to horizontal.

In our study, the mean RMP of LC neurons in morphine-dependent group in the presence of continuous 5 μM morphine were -54.2 ± 2.6, -53.4 ± 1.4 and -54.3 ± 2.9 mV for HZ, OTH-cut and OTH brain slices, respectively (Fig 4A–4C). A clear depolarization in RMP was observed in these slices after naloxone treatment. The average RMP in LC neurons following naloxone superfusion in morphine-dependent group were -50 ± 3, -49.3 ± 3 and -48.1 ± 1.9 mV for HZ, OTH-cut and OTH brain slices, respectively (Fig 4A–4C). In addition, LC neurons from dependent animals exhibited a higher mean SDR after naloxone application than that of naïve rats, which was evident in all three slice preparations (Fig 2 versus Fig 4).

Moreover, as shown in Fig 4D, the net effect of naloxone on RMP and SDR in OTH brain slices was significantly (p < 0.05) higher than those of HZ and OTH-cut brain slices of morphine dependent rats. Net RMP depolarization were 4.2 ± 0.5, 4.1 ± 0.4 and 6.2 ± 0.4 mV and net increased SDR were 0.7 ± 0.14, 0.7 ± 0.2 and 1.2 ± 0.1/s for HZ, OTH-cut and OTH brain slices, respectively.

### Effect of kynurenic acid on withdrawal-induced hyperactivity of LC neurons in morphine dependent rats

**Fig 5:**
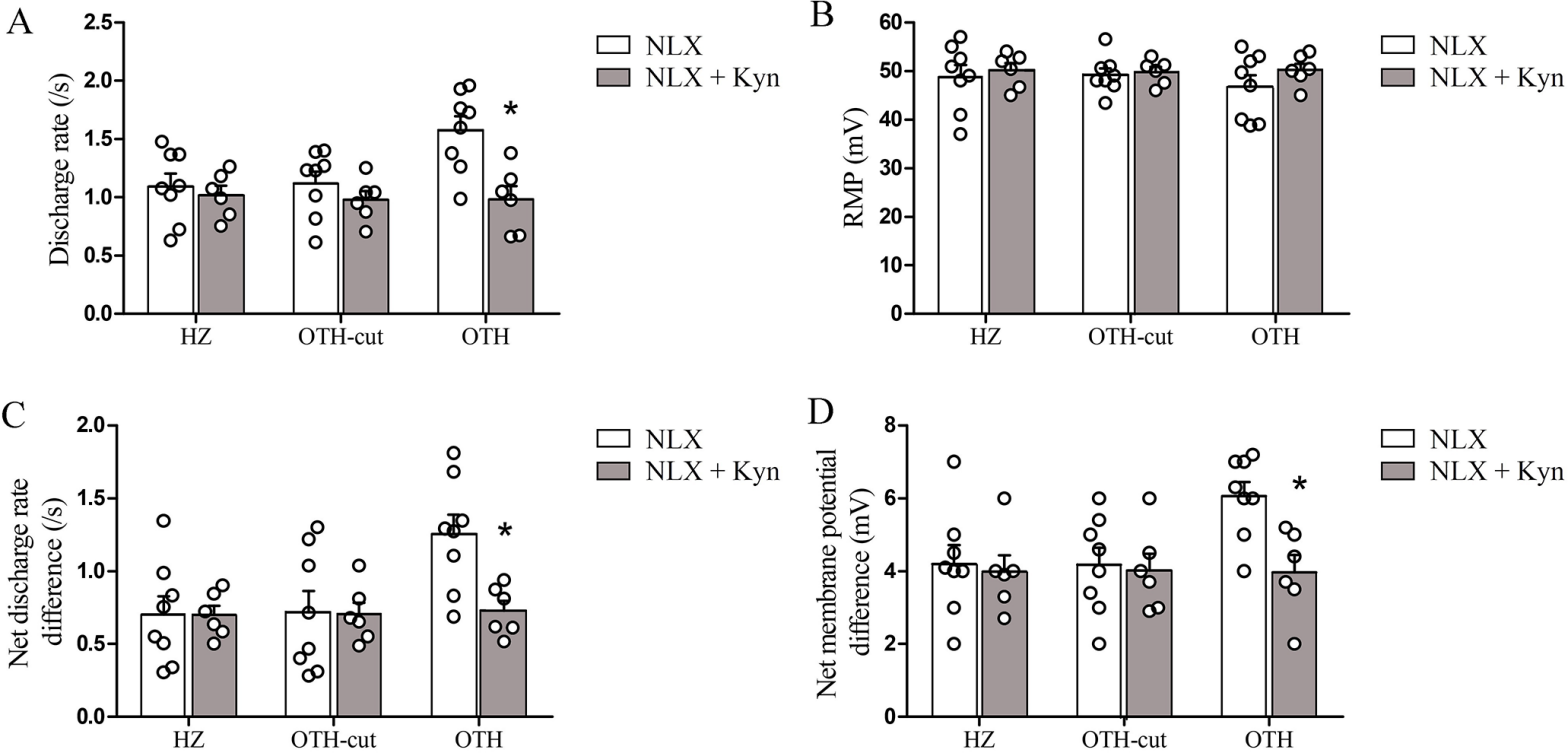
Effect of kynurenic acid on LC neuronal activity following morphine withdrawal in morphine-dependent rats. Summary data showing the effect of kynurenic acid (500 μM) on the SDR (A: total and C: net) and RMP (B: total and D: net) of LC neurons following opiate withdrawal in all three slice forms taken from morphine treated rats. Kynurenic acid application in HZ and OTH-cut brain slices did not significantly affect the net increased SDR and RMP values following withdrawal-induced hyperactivity of LC neurons. Also, Treatment of OTH brain slices with kynurenic acid deceases the observed increment in LC neuronal SDR and RMP in comparison to HZ and OTH-cut brain slices. Data are expressed as mean ± SEM, n = 6–8 in each type of brain slice, * P < 0.05 versus LC neuronal SDR and RMP from OTH brain slices without kynurenic acid application. Data were analyzed using One-way ANOVA followed by Tukey’s post hoc test. Kyn: kynurenic acid; RMP: resting membrane potential; SDR; spontaneous discharge rates; NLX: naloxone; LC: locus coeruleus; HZ: horizontal; OTH-cut: oblique to horizontal-cut; OTH: oblique to horizontal.

In order to investigate the effect of PGi excitatory afferents on naloxone-induced hyperactivity of LC neurons in chronically morphine treated rats, kynurenic acid (an excitatory amino acid antagonist) was used. As shown in Fig 5C and 5D, kynurenic acid (Kyn) administration (500 μM) attenuated the observed net increment in LC neuronal SDR and RMP to that of receiving no PGi inputs (HZ and OTH-cut brain slices). Also, kynurenic acid application in HZ and OTH-cut brain slices did not significantly affect the increased SDR and RMP values following withdrawal-induced hyperactivity of LC neurons (Fig 5).

### Frequency and amplitude of spontaneous EPSCs in naïve rats

**Fig 6:**
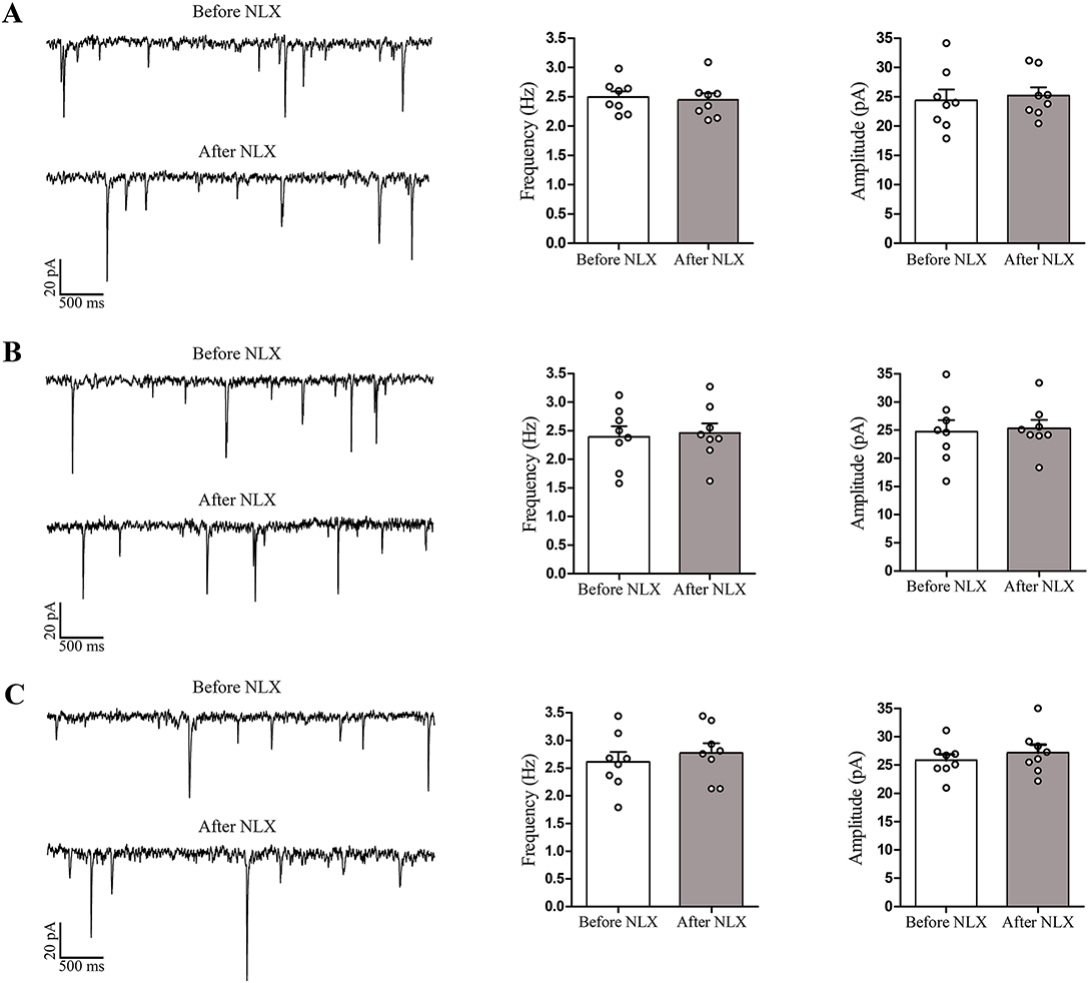
Frequency and amplitude of sEPSCs in LC neurons of naïve (non-dependent) rats. The samples traces of sEPSCs before and after application of naloxone (1 μM) in LC neurons of HZ (A), OTH-cut (B) and OTH (C) brain slices taken from naïve rats. Histograms indicate the mean frequency and amplitude of sEPSCs recorded before and after naloxone application. No significant alteration was observed in amplitude and frequency of sEPSCs in LC neurons of all slice forms following naloxone application. Data are expressed as mean ± SEM, n = 8 in each type of brain slice. Data were analyzed using paired Student’s t-test. NLX: naloxone; LC: locus coeruleus; sEPSCs: spontaneous excitatory post-synaptic currents; HZ: horizontal; OTH-cut: oblique to horizontal-cut; OTH: oblique to horizontal.

As shown in Fig 6, frequency of sEPSCs as well as amplitude index were not significantly different among three forms of brain slices taken from naïve rats. Furthermore, application of naloxone did not alter the frequency and amplitude of sEPSCs in LC neurons of all three slice forms in naïve rats (Fig 6A–6C).

### Frequency and amplitude of spontaneous EPSCs in morphine dependent rats

**Fig 7:**
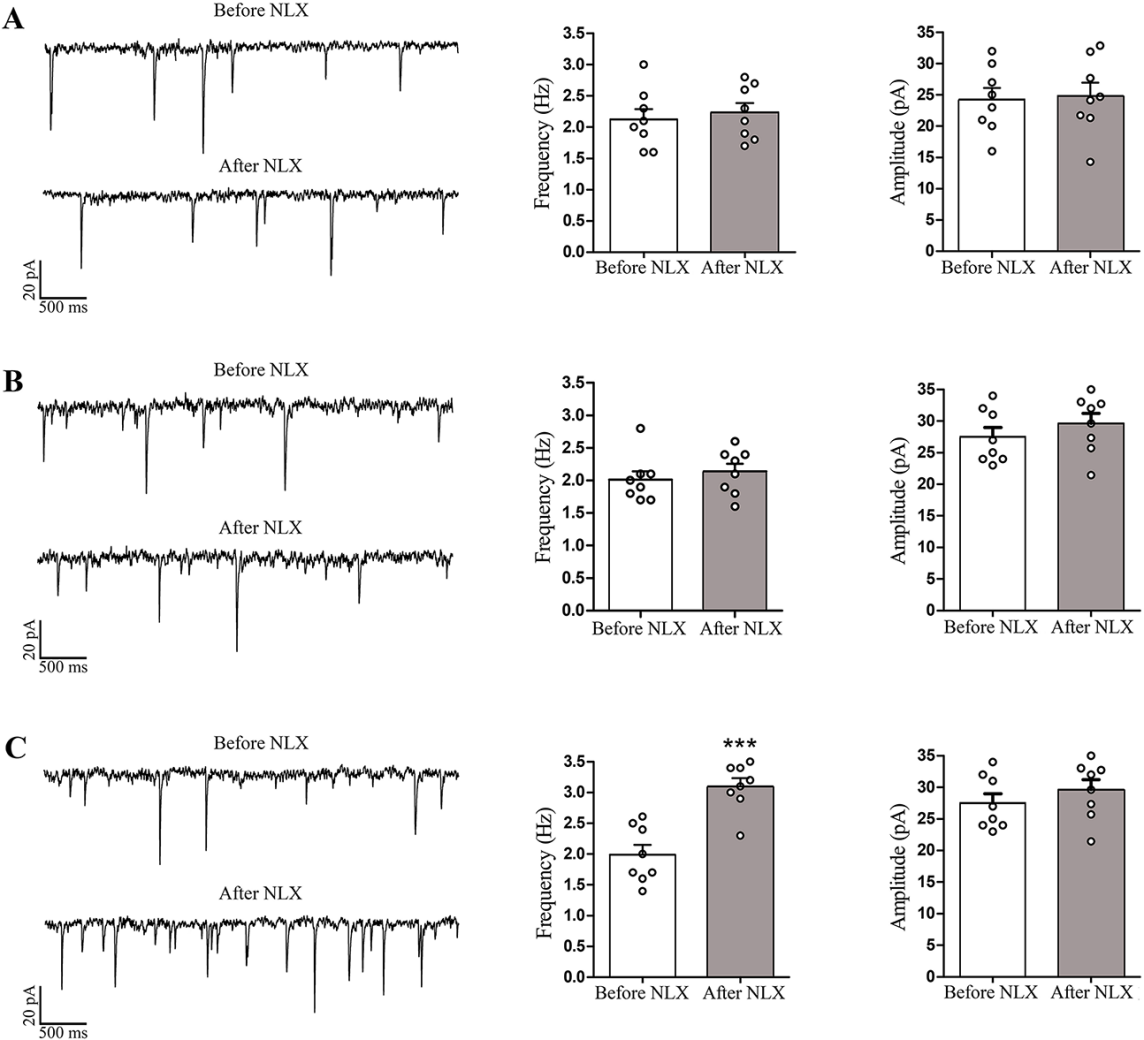
Effect of naloxone on frequency and amplitude of EPSCs in LC neurons of morphine-dependent rats. The samples traces of sEPSCs before and after application of naloxone (1 μM) in LC neurons of HZ (A), OTH-cut (B) and OTH (C) brain slices taken from morphine dependent rats. The slices were bathed in 5 μM morphine. There was no significant alteration in amplitude and frequency of sEPSCs in LC neurons of HZ and OTH-cut slices following naloxone application. As shown in histograms, the frequency of sEPSCs in LC neurons of OTH brain slices has significantly increased following naloxone application. However, no significant change was observed in the amplitude of sEPSCs after naloxone treatment in OTH brain slices. Histograms show the mean frequency and amplitude of sEPSCs recorded before and after naloxone application. Data are expressed as mean ± SEM, n = 8 in each type of brain slice, *** P < 0.001 versus before naloxone application. Data were analyzed using paired Student’s t-test. NLX: naloxone; LC: locus coeruleus; sEPSCs: spontaneous excitatory post-synaptic currents; HZ: horizontal; OTH-cut: oblique to horizontal-cut; OTH: oblique to horizontal.

In the present study, though the naloxone superfusion increased the mean frequency of the sEPSCs in LC neurons significantly (P < 0.001) from 1.9 ± 0.2 to 3.1 ± 0.3/s in OTH slices taken from morphine treated rats (n = 8), but this treatment did not significantly affect its mean amplitude (28.3 ± 3.7 versus 31.8 ± 4.7 pA), as shown in Fig 7C. However, naloxone altered neither the frequency nor the amplitude of sEPSCs in LC neurons of HZ and OTH-cut brain slices (Fig 7A and 7B).

## Discussion

Identification of the neuronal network mediating autonomic, neurochemical and behavioral responses are essential for understanding opioid dependence and withdrawal. Among these diversified networks strong projections from PGi to LC have been verified by various anterograde and retrograde neuronal tracers and these routes have been confirmed via antidromic electrophysiologic stimulations [21].

In this study, we developed an oblique to horizontal (OTH) brain slice containing both the LC and PGi neurons (Fig 1). The existence of both brain regions was detected using HRP retrograde neuronal tracing in this slice preparation. Moreover, the OTH slice was prepared in a way to contain at least some PGi to LC projections within its 400 μm thickness, which was further verified by applying a low intensity, single electrical pulse to PGi neurons followed by recording of evoked EPSCs from LC neurons (mean latency of evoked EPSCs were 5.12 ± 0.2 ms; Fig 1A, right side).

It has been shown by retrograde neuronal tracing that the PGi nucleus, located in RVLM, provides the main LC afferents [27]. It has also been reported that there might be at least three distinct projecting pathways from PGi to LC- first one climbs dorsally along the lateral sides of the pons, then turns to reach the LC nucleus along the lateral-to-medial axis, the second pathway is located in the medial part of medulla, ventral to the medullary adrenergic bundle and the third one is the ascending adrenergic pathway innervating the LC nucleus [20]. Our results confirm the existence of some functional connections between PGi and LC regions within the OTH slices. With respect to aforementioned pathways, the first as well as the second ones are more probable to provide the main afferents from PGi to LC in our OTH slices, as the orientation of cutting stage (Fig 1B and 1C) was in line with these anatomical routes.

In addition, since PGi to LC connecting bundles were cut in OTH-cut slices, electrical stimulation of PGi region did not result in evoked EPSCs in LC neurons. So, LC neurons have not been affected by intact connecting fibers from PGi in this type of slice preparation. Also, prepared HZ slices had no PGi region, thus the presence of intact PGi to LC afferents would not be possible.

Our findings indicated that following acute morphine administration in brain slices taken from non-dependent animals, SDR was suppressed and RMP decreased to more hyperpolarized values in LC neurons compared to those of slices taken from naïve (untreated) rats (Fig 2 versus Fig 3). However, SDR and RMP values returned to the level of LC neurons taken from naïve animals following naloxone application in acute morphine group (Fig 3). Our results are very much consistent with previously reported findings [8].

It is noteworthy that, before naloxone application, SDR and RMP values in LC neurons of chronically morphine treated rats of our study were maintained around those of naïve animals (Fig 2 versus Fig 4), which was due to development of tolerance to morphine in LC neurons of dependent animals. In this regard, it has been reported that, opiate binding to μ opioid receptors (MORs) results in decreased activity of adenylyl cyclase (AC) and cAMP signaling [10]. Also, acute binding of opiates to the MORs reduces the spontaneous activity of LC neurons, primarily by activating G protein-gated inwardly-rectifying K^+^ (GIRK) channels [28,29]. In addition, following long term opiate treatment, both firing rate and cAMP signaling return to the baseline level due to an up-regulation of cAMP pathway representing tolerance to opiates [5,13,30].

In the present study, during naloxone-induced withdrawal, the SDR of LC neurons increased significantly compared to the control group in HZ and OTH-cut brain slices (that is, lacking intact PGi afferents) taken from morphine dependent rats (Fig 2 versus Fig 4). It should be noted that following naloxone application, no significant difference was observed in RMP between the HZ and OTH-cut brain slices of control and morphine dependent rats.

These findings are consistent with the previous study report indicating that the LC neuronal discharge rate increased following naloxone administration in morphine dependent rats while the RMP did not become significantly more positive than the RMP value in naïve animals [8]. This means that similar to the previously reported in vivo investigations, LC neurons could show the local withdrawal-induced hyperactivity in vitro [31]. Also, in this part of the study results are in line with previous in vitro extracellular study which indicated in brain slices taken from morphine dependent rats, LC neuronal discharge rate increases in response to opioid withdrawal [30]. It has also been reported that intra-LC microiontophoretic administration of naloxone (as an opiate antagonist) increases LC neuronal activity in morphine dependent rats [5].

It is noteworthy that the previously reported withdrawal-induced hyperactivity of LC neurons could not be completely abolished by antagonizing excitatory amino acid (EAA) transmission or lesions of LC afferents [22,32]. Furthermore, it has been demonstrated that a two fold increase in LC firing rate was observed following opioid receptor antagonism in cultured LC-containing slices (coronal section from rat brain) which had been chronically treated with morphine [7]. Ivanov and Aston-Jones (2001), using quasihorizontal brain slices, (partly similar to HZ slices and without PGi region) have shown that withdrawal-induced hyperactivity of LC neurons taken from morphine dependent rats could only be affected to somehow by glutamate and GABA receptor antagonists or by tetrodotoxin (TTX) administration (in order to prevent synaptic transmission). In other words, these manipulations were unable to inhibit the increased activity of LC neurons [8]. Thus, only a small part of withdrawal-induced hyperactivity of LC neurons might be mediated by EAA inputs at in vitro condition. This phenomenon seems to be occurred at the result of LC neuronal adaptations to chronic morphine treatment. However, previous investigations have unveiled to some extent that the molecular mechanisms involved in this neuronal adaptation, where chronic morphine administration resulted in up-regulation of AC I, AC VIII, cAMP, CREB and protein phosphorylation pathways in LC neurons [3,11,33,34]. In addition, both the increased activity of LC neurons and the intensity of withdrawal-induced behavioral features were decreased by blocking of any of proposed molecular pathways [7,35].

In the present study it was shown that LC neurons receiving intact excitatory synaptic inputs from PGi region in OTH brain slices displayed a higher net discharge rate during opiate withdrawal compared to the LC neurons in HZ or OTH-cut preparations (Fig 4D). Also, the net resting membrane potential changes before and after naloxone administration in LC neurons of OTH brain slices was significantly more positive than those of LC neurons receiving no intact excitatory synaptic inputs from PGi (HZ and OTH-cut brain slices). It seems that, PGi excitatory afferents may increase LC neuronal discharge rate during naloxone induced opiate withdrawal.

Our findings about the effect of kynurenic acid on the three types of brain slices taken from morphine dependent rats (Fig 5), also support the idea that increase in net SDR and RMP values of LC neurons in OTH slices (compared to other brain slice types) following naloxone application might be due to the increased activity of PGi excitatory afferents. In addition, the partial attenuating effect of kynurenic acid on SDR and RMP of LC neurons in OTH slices and ineffectiveness of this EAA antagonist on the same variables SDR and RMP in HZ and OTH-cut slices following naloxone administration revealed that, observed increment in excitability indices of LC neurons in OTH slices could not be attributed solely to the neurochemical changes induced during development of opioid tolerance.

An in vivo microdialysis study has confirmed the involvement of other brain regions in opiate withdrawal-induced hyperactivity of LC neurons, where the extracellular release of glutamate and aspartate in LC nucleus was increased during opiate withdrawal [19].

It has also been reported that intracerebroventricular (i.c.v.) administration of kynurenic acid prior to opiate antagonist injection, prevented withdrawal-induced hyperactivity of LC neurons [36]. Since LC nucleus receives afferents primarily from two brain regions-PGi in RVLM and nucleus prepositus hypoglossi (PrH) in dorsomedial rostra1 medulla [20,27], so, these areas seem to be the main candidates for mediating the extrinsic modulation of LC neuronal activity during opiate withdrawal. However, as PrH nucleus sends strong GABAergic projections to LC, most likely it might not play a critical role in hyperactivity of LC neurons during opiate withdrawal. Instead, LC receives strong excitatory inputs from PGi which could mediate in part withdrawal-induced hyperactivity of LC neurons [12].

It has also been shown that low intensity, single-pulse electrical stimulation of PGi resulted in excitation of approximately 73% of LC neurons [21]. Regarding the involvement of excitatory afferents of PGi in mediating withdrawal induced hyperactivity of LC neurons, previous studies have reported that following lesions of PGi (as the main LC glutamatergic input) or i.c.v. microinjection of kynurenic acid, there was decrement in withdrawal-induced hyperactivity of LC neurons [22,36]. However, in our study, the frequency of sEPSCs recorded from LC neurons in OTH brain slices has significantly increased following naloxone application, though the amplitude was not affected after the same treatment in the same slices. It is noteworthy that the frequency of sEPSCs in HZ and OTH-cut slices did not significantly increase after naloxone administration in morphine dependent animals (Fig 7).

Moreover, different in vivo extracellular recordings have shown that the discharge rate of PGi neurons in morphine dependent rats significantly increases following naloxone administration compared to the control (non-dependent) rats [37–39]. Thus, it is possible that the observed increment in LC neuronal activity following opiate withdrawal might be due to increment in the activity and therefore the excitatory tone of PGi neurons.

## Conclusion

In conclusion, our results strengthen the idea that intact PGi inputs, preserved in the OTH slices, facilitate the cellular expression of morphine withdrawal in the LC neurons. This is further supported by the electrophysiological differences observed among slice preparations. However, such facilitative effect of intact PGi afferents on cellular expression of morphine withdrawal in LC neurons has not been directly investigated in vitro. Also, these findings show that the hyperactivity of LC neurons during opiate withdrawal might be due to an increment in their resting membrane potential. This in turn could be mediated by an increase in the excitatory tone of PGi neurons to LC.
